# Not Waiting to Progress; How the COVID-19 Pandemic Nudged Neoadjuvant Therapy for Stage III Locally Advanced Melanoma Patients

**DOI:** 10.3390/curroncol30050335

**Published:** 2023-04-23

**Authors:** Katie Kinaschuk, Tina Cheng, Thomas Brenn, J. Gregory McKinnon, Claire Temple-Oberle

**Affiliations:** Tom Baker Cancer Centre, 1331-29 Street NW, Calgary, AB T2N 4N2, Canada

**Keywords:** neoadjuvant therapy, melanoma, COVID-19

## Abstract

*Background*: Early-phase neoadjuvant trials have demonstrated promising results in the utility of upfront immunotherapy in locally advanced stage III melanoma and unresected nodal disease. Secondary to these results and the COVID-19 pandemic, this patient population, traditionally managed through surgical resection and adjuvant immunotherapy, received a novel treatment strategy of neoadjuvant therapy (NAT). *Methods*: Patients with node-positive disease, who faced surgical delays secondary to COVID-19, were treated with NAT, followed by surgery. Demographic, tumour, treatment and response data were collected through a retrospective chart review. Biopsy specimens were analysed prior to the initiation of NAT, and therapy response was analysed following surgical resection. NAT tolerability was recorded. *Results*: Six patients were included in this case series; four were treated with nivolumab alone, one with ipilimumab and nivolumab and one with dabrafenib and trametinib. Twenty-two incidents of adverse events were reported, with the majority (90.9%) being classified as grade one or two. All patients underwent surgical resection: three out of six patients following two NAT cycles, two following three cycles and one following six cycles. Surgically resected samples were histopathologically evaluated for the presence of disease. Five out of six patients (83%) had ≤1 positive lymph node. One patient showed extracapsular extension. Four patients demonstrated complete pathological response; two had persisting viable tumour cells. *Conclusions*: In this case series, we outlined how in response to surgical delays secondary to the COVID-19 pandemic, NAT was successfully applied to achieve promising treatment response in patients with locally advanced stage III melanoma.

## 1. Introduction

The COVID-19 pandemic was a time of unprecedented challenges that impacted nearly all individuals and sectors. Healthcare workers and the healthcare sector were hit particularly hard, confronting various challenges in resource allocation, everchanging staffing and work protocols, as well as limited access and delays to various services and treatments [1].

At the Tom Baker Cancer Centre in Calgary, Canada, patients with locally advanced stage III melanoma and unresected nodal disease have been traditionally managed with initial surgical resection and 12 cycles of subsequent adjuvant therapy [2,3,4,5,6,7,8]. This adjuvant therapy is either immunotherapy or targeted, depending on tumour BRAF status and clinician and patient preferences. Profound delays in OR access, secondary to the COVID-19 pandemic, created a surgical crisis at our centre, like many others [9]. During the fourth wave of the pandemic, operating room (OR) wait times for these surgical cases superseded 8 weeks, which was already double the recommended intervention timeframe [10]. This delay occurred because melanoma patients are categorized to have category B cancers, and during the depths of the pandemic, OR access was limited to patients with imminently life-threatening category A cancers. With data extrapolation predicting a normalization of OR access in approximately 2.5 months’ time, this, along with the backlog and already stretched resources of the Canadian healthcare system, were anticipated to continue to act as barriers to care for these patients.

In response to this challenge, and in the spirit of innovation and patient care, our leadership and tumour group, through careful planning and execution, advocated for a temporary deviation from traditional management for patients at high risk of adverse outcomes with surgical delay [11,12]. This granted a small subset of patients, as presented in this case series, access to a deviated treatment protocol that was planned to be consistent with those of favourable literature findings [13,14,15,16].

Our emergency COVID response novel treatment strategy was in line with promising neoadjuvant therapy (NAT) early-phase trials. These trials, such as OpACIN, OpACIN-neo and PRADO, have demonstrated promising results in patients with locally advanced stage III melanoma and unresected nodal disease [13,14,15,16,17]. In these and other trials, the utility of upfront immunotherapy demonstrated favourable response-directed surgical management and outcomes [18]. As such, this emergency authorization saw patients with stage III melanoma and regional nodal disease with a metastatic burden greater than 1 mm, who were considered to have a poor prognosis, be eligible for 12 cycles of combined neoadjuvant and adjuvant therapy consisting of anti-PD1 monotherapy or dabrafenib/trametinib [2,3,4,5,6,7,8]. These expedited adjuvant therapy cycles were to be delivered preoperatively as NAT, with the remaining cycles delivered post-operatively.

We recognized that NAT is suitable for clinical trials only and ensured that postoperative adjuvant therapy was not response driven—all patients were scheduled for a total of 12 cycles of therapy. Approval of this novel therapeutic management ultimately allowed for the administration of NAT:

1. To patients with stage III melanoma and gross nodal disease who were deemed unresectable, in that they cannot be resected due to an expected OR delay of ≥8 weeks;
2. With discussion and approval in multidisciplinary rounds;
3. With an audit of outcomes.

This case series discusses a single-site experience of the utilization of neoadjuvant administered systemic therapy in such patients and comprises the audit of outcomes. 

## 2. Materials and Methods

All sex patients, 18 years of age and older, with locally advanced stage III melanoma and unresected nodal disease, who were being treated at the Tom Baker Cancer Centre and experienced a delay in surgical resection greater than 8 weeks, secondary to the fourth wave of the COVID-19 pandemic (October 2021 to January 2022), were included in this case series. Six such patients were identified by the Tom Baker Cancer Centre cutaneous group and were subsequently deemed to be at high risk for disease progression and poor outcomes given this surgical delay. 

This subset of patients was alerted to the proposed non-traditional timing of drug therapy in their disease setting and given the rationale for this treatment protocol deviation. Patients who agreed to this deviation were provided with NAT. For inclusion in this case series, each patient signed a consent form agreeing to the release and analysis of their pertinent medical information, as well as for publication in the medical literature. 

Data for this case series were collected prospectively and analysed retrospectively via chart review. Data, including demographics, NAT type and the number of preoperative cycles, NAT-associated adverse events (AEs), and histopathological analysis of surgical specimens, were extracted into a locked spreadsheet and coded for anonymity.

Prior to the initiation of NAT, biopsy samples were analysed to confirm the diagnosis of melanoma, as well as to determine the BRAF mutation status. Disease response of surgically resected specimens was subsequently analysed and reported according to the International Neoadjuvant Melanoma Consortium meetings in 2016 and 2017 guidelines for pathologic examination and reporting of surgical specimens from AJCC (8th edition) stage IIIB/C/D treated with neoadjuvant-targeted or immunotherapy guidelines [19].

The tolerability of NAT was assessed according to Common Terminology Criteria for Adverse Events, Version 5 [20].

## 3. Results

Table 1 describes the basic demographic data of each patient, as well as their disease status and NAT regimen. Prior to the initiation of NAT, each patient had their disease burden assessed through biopsy analysis and a PET scan. 

Four out of six patients included were ≥60 years. Two-thirds were female. Three patients were BRAF (+); two had a V600E mutation, and one a V600R mutation. No patient had evidence of systemic disease, as per negative PET scans, but one patient had lung nodules and a left supraclavicular LN of unknown significance identified. 

Patients received neoadjuvant immunotherapy consisting of ipilimumab and/or nivolumab or targeted therapy with dabrafenib and trametinib. Four out of six patients were treated with nivolumab alone. One patient was treated with a combination of ipilimumab and nivolumab, and one patient was treated with dabrafenib and trametinib. Half of the patients (three out of six) received two neoadjuvant cycles before surgery, with two patients receiving three, and one patient reassigned from surgical management to continue further cycles secondary to an excellent response to dabrafenib and trametinib, who later underwent surgical management following 6 months of continuous NAT. Overall, all patients underwent surgical resection, and all patients were to receive a total of 12 cycles of systemic therapy.

Table 2 outlines the 22 reported treatment-related AEs. The AEs ranged in severity from grade one to three, with the majority (90.9%) of AEs being classified as grade one or two. The most commonly reported AEs were rash (four patients) and fatigue (three patients). Hypophysitis occurred in one patient -treated with a combination of ipilimumab and nivolumab- as a grade three AE. Notably, the only AE associated with all three treatment regimens was a rash.

Following surgical resection, a dermatopathologist assessed the excised tumour tissue and lymph nodes (LNs) for disease response, defined as treatment-related changes (TRCs). These changes are reflective of neoadjuvant therapeutic success in targeting malignancy. Observed TRCs include histopathological markers of cystic degeneration, fibrosis, the presence of inflammatory cells and melanophages, as well as scattered pigment. Table 3 outlines the oncologic outcomes of disease response following NAT, presenting the characteristics of excised LNs, including the number of positive LNs, the location from which they were resected, extracapsular extension, and tumour viability.

**Figure 1:**
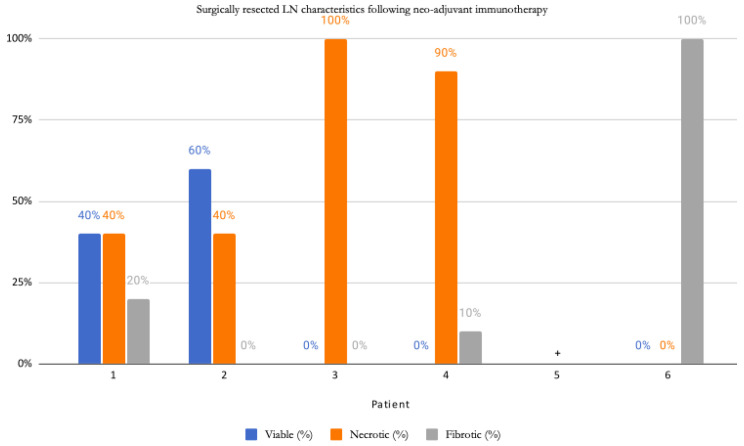
Outlines surgically resected LN characteristics, including viability, necrosis, and fibrosis, following NAT. ⁺ No metastasis or treatment effect was detected by dermatopathology, but some fibrotic changes were reflective of anticipated changes to a prior biopsy site; LN—lymph node.

Five out of six patients had ≤1 positive LNs detected. Of these, four out of five were found to be absent of extracapsular extension. Overall, four of the six patients had a pathological complete response (pCR), and two had a partial response with a 40–60% persistence of viable tumour cells (Figure 1).

**Figure 2:**
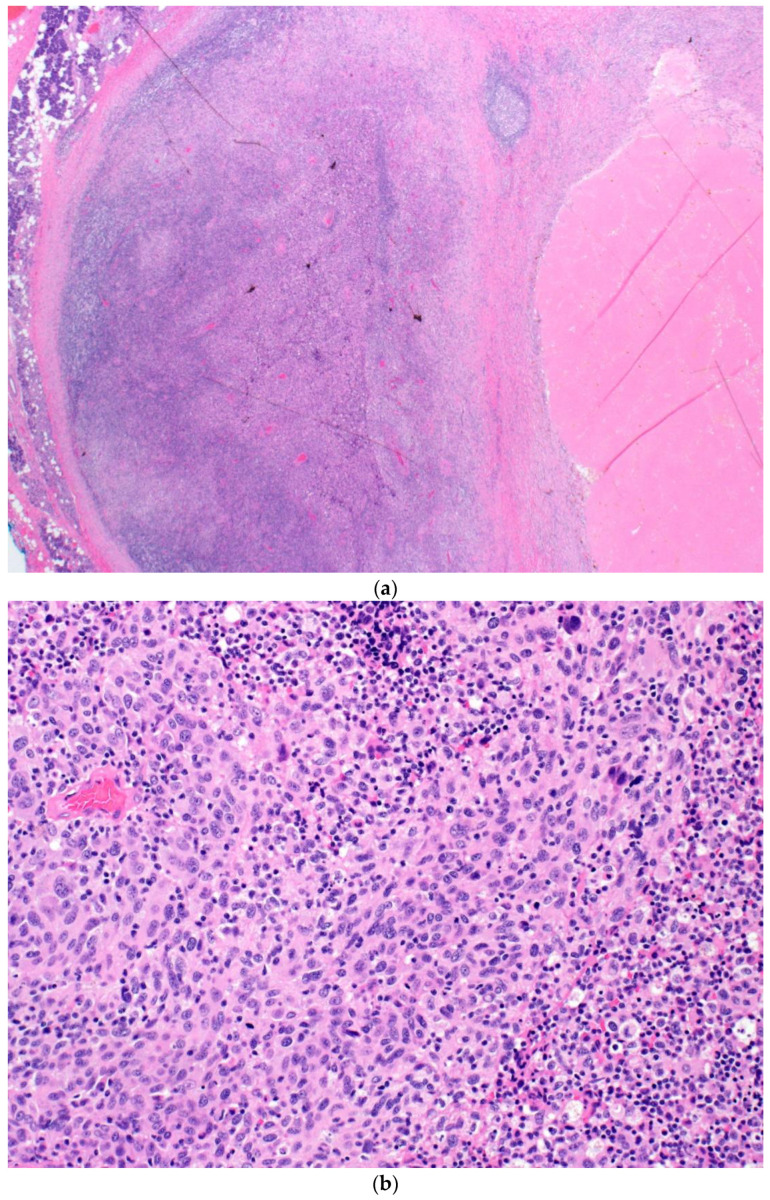

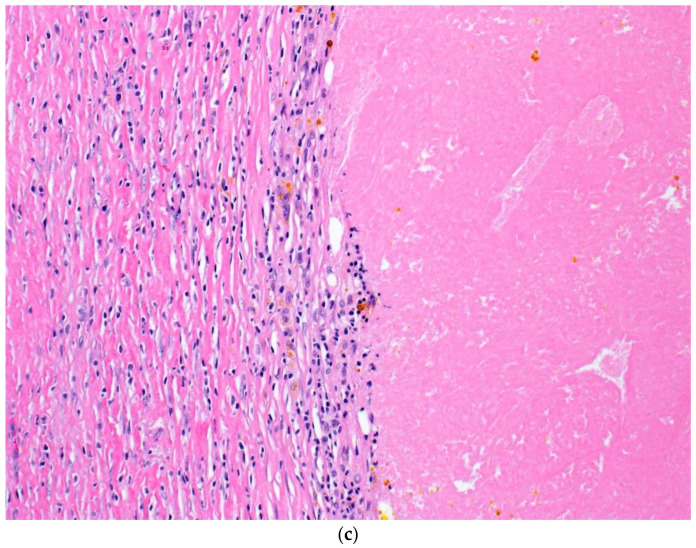
(**a**) A low-power shot at 10× magnification showing the intraparotid LN with areas of tumour necrosis on the right. (**b**) A high-power shot showing the intraparotid LN with areas of residual viable melanoma. (**c**) A high-power shot showing the intraparotid LN with areas of fibrosis and tumour necrosis.

Figure 2 shows images taken from a surgically resected intraparotid LN sample from patient one, demonstrating treatment response and residual viable tumour cells

## 4. Discussion

The COVID-19 pandemic was a time of unprecedented challenges that demanded novel and timely solutions. In this case study, we outlined the novel use of NAT in the treatment of locally advanced stage III melanoma and unresected nodal disease. 

Secondary to the fourth wave of the COVID-19 pandemic, patients with locally advanced stage III melanoma and unresected nodal disease being managed at the Tom Baker Cancer Centre in Calgary, Canada, experienced delays in access to surgical management due to limited OR resources. The currently accepted standard of care sees these patients undergoing surgical resection within a 4-week timeframe from diagnosis [10]. With the pandemic-related restrictions delaying surgical management past the 8-week mark, there was a concern for disease progression and increased morbidity and mortality. Consequently, the opportunity for NAT was pursued in this subset of patients, who were ultimately granted emergency use of NAT based on promising literature on the subject [13,14,15,16,17,21,22].

Early-phase OpACIN (NCT02437279) -phase Ib- and OpACIN-neo (NCT02977052) -phase II- clinical trials have shown high pathologic response rates in patients with macroscopic stage III melanoma receiving neoadjuvant ipilimumab plus nivolumab [13,14,15]. After 4 years, none of the patients with a pathologic response (*n* =  7/9 patients) in the OpACIN study had relapsed [15]. In OpACIN-neo (*n* =  86), the 2-year estimated relapse-free survival (RFS) was 84% for all patients, 97% for patients achieving a pathologic response and 36% for nonresponders (*p* <  0.001) [14].

In addition, a pooled analysis from six neoadjuvant clinical trials for stage III melanoma, including 192 patients (141 receiving immunotherapy and 51 receiving targeted therapy), showed that 40% of patients had a pCR [16]. This pCR correlated with improved RFS (2-year RFS with pCR was 89%) and overall survival (OS) (2-year OS was 95%). This improved RFS and OS was especially noted in patients treated with immunotherapy, where very few relapses were identified [16].

In this case series, we reported on six patients who received NAT; four out of six patients were treated with nivolumab alone, one patient was treated with a combination of ipilimumab and nivolumab, and one patient was treated with dabrafenib and trametinib (Section 3, Table 1). This treatment was well-tolerated by patients, with 90.9% of the twenty-two reported AEs being classified as grade one or two. 

All patients underwent surgical resection when OR access was available. Three out of six patients underwent surgical resection following two neoadjuvant cycles, two patients following three cycles, and one patient following six cycles. The last patient had their OR further delayed secondary to excellent NAT treatment response. All patients were scheduled to receive a total of 12 cycles of immunotherapy.

Surgically resected samples from all patients demonstrated treatment response, as per histopathological analysis. Four out of six patients demonstrated pCR. Eighty-three percent of patients (five out of six) had ≤1 positive LN, with only one patient showing extracapsular extension. 

Although our case series is limited to a timeframe <1 year, we are hopeful that as all patients were treatment responders, with four out of six patients showing pCR, these patients will experience improved RFS and OS overtime, as reflective of the evidence demonstrated in the literature [14,15,16].

In addition, a neoadjuvant approach may not only improve OS but also, as seen in our case series, reduce advanced nodal presentations, allowing for surgical resections with less morbidity. This has been seen in other tumour types [21,22].

Data from other tumour types, as well as the results from this case series, demonstrate that the use of NAT can mitigate patient care in times of delayed access to treatment. The use of NAT can also be helpful in the assessment of therapy response, which can include the following:

1. When the response to therapy is as anticipated;
2. When the response to therapy is limited;
3. When the response to therapy supersedes expectations.

When the response to therapy is as anticipated, i.e., mitigating tumour load, surgical resection can be planned. As demonstrated in this case series, histopathology of surgically resected tissues often demonstrated a good treatment response, with few positive LNs and limited tumour viability, with most of the tumour tissue being classified as fibrotic and/or necrotic, reflective of limited tumour spread. Alternatively, when the response to therapy is limited, alternative therapeutic options and overall management can be reviewed to limit patient morbidity, i.e., considering foregoing surgical intervention when the response to systemic therapy has been limited. Conversely, as seen in patient six of this case series, the use of NAT has the potential to downstage tumours, allowing for more conservative surgical management options [23]. Not only does this benefit the patient in reducing surgery-associated morbidity and risk, but it also utilizes fewer health system resources [21,22,23]. Overall, the results from our six-patient series are promising in demonstrating the possible benefits of utilizing NAT in this patient cohort. 

Limitations to the case series include the small number of individuals included and a period of data collection limited to a timeframe from October 2021 to May 2022, with no long-term follow-up data on morbidity or possible survival benefit. All patients, however, are alive to date. 

## 5. Conclusions

In this case series, we outlined how the novel use of NAT in the treatment of locally advanced stage III melanoma and unresected nodal disease in a time of delayed access to surgical resection secondary to the COVID-19 pandemic was an effective and acceptably tolerated therapeutic management strategy, with all patients demonstrating treatment response, and the majority demonstrating complete response, as per histopathology analysis. We believe our results demonstrate the potential benefits of utilizing NAT in this patient cohort and will contribute to the evolving body of evidence on this topic. 

## Figures and Tables

**Table 1:** Outlines the demographic and disease status of each patient, as well as NAT regimen.

Patient	Age	Sex	Primary Site	BRAF Status; Variant	Evidence of Systemic Disease	Locally Advanced Presentation	NAT Agent(s)	NAT Cycles Completed	Time from Last NAT to Sx (Days)	Post-Sx NT (# of Cycles)
1	60	M	L temple	(+) V600E	None	L parotid adenopathy	ipilimumab + nivolumab	3	20	10
2	62	M	L tragus	(+) V600R	None	L parotid adenopathy	nivolumab	2	20	10
3	27	F	R ear	(−)	None	R parotid and neck adenopathy	nivolumab	2	41	10
4	65	F	midline back	(−)	None	R axillary adenopathy	nivolumab	2	48	10
5	65	F	L posterior neck	(−)	None	L neck adenopathy	nivolumab	3	28	9
6	55	F	L distal. medial thigh	(+) V600E	Lung nodules and L supraclavicular LN *	L thigh mass > 10 cm (20 cm of surrounding skin discolouration)	dabrafenib + trametinib	5	0 ^†^	7

F, female; L, left; LN, lymph node; M, male; NAT, neoadjuvant treatment; R, right; Sx, surgery; (−), negative; (+), positive; * this finding was determined to be of unknown significance; ^†^ the patient was dosing her NAT on a continuous daily basis, as directed.

**Table 2:** Outlines the incidence of reported treatment-related AEs and percentage graded 3/4.

Treatment-Related Adverse Event	Incidence	Grade 3–4 (%)	Associated NAT (Y/N)		
			Nivolumab Alone (*n* = 4)	Ipilimumab and Nivolumab (*n* = 1)	Dabrafenib and Trametinib (*n* = 1)
Fatigue	3 *	0	Y	Y	N
Insomnia	1	0	Y	N	N
Rash	4	0	Y	Y	Y
Pruritus	1	0	Y	N	N
Nausea	2	0	Y	Y	N
Abdominal cramping	1	0	N	Y	N
Diarrhea	1	0	Y	N	N
Arthritis	1	0	Y	N	N
Increased TSH	1	0	Y	N	N
Headache ⁺	1	0	Y	N	N
Dyspnea ⁺	1	0	Y	N	N
Multisystem inflammatory syndromes	1	0	N	Y	N
Alopecia	1	0	N	Y	N
Hypophysitis and related symptoms ^†^	2	100%	N	Y	N
Pancreatitis	1	0	N	Y	N

* one patient reported fatigue twice due to worsening severity; ⁺ secondary to thyroid storm; ^†^ one patient reported two incidents of hypophysitis and related symptoms.

**Table 3:** Outlines the characteristics of lymph nodes and tissue following surgical resection.

Patient	Procedure	# of (+) LNs/Total LNs	Size of Largest Metastatic Deposit in LN	Location of (+) LN	Extracapsular Extension (Y/N)	Response
1	L parotidectomy, neck dissection	1/58	6 mm	parenchymal	N	PR
2	Wide excision melanoma L cheek, L parotidectomy, L functional neck dissection	6/20	5 mm	parenchymal	N	PR
3	R parotidectomy, R neck dissection	1/32	10 × 7 mm	parenchymal	Y	CR
4	R axillary LN dissection	1/23	8 × 7 mm	parenchymal	N	CR
5	L modified neck dissection	0/29	NA	NA	N	CR
6	L thigh	0	NA	Dermis and subcutaneous tissues	N	CR

CR, complete response; L, left; LN, lymph node; MM, metastatic melanoma; mm, millimetre; NA, not applicable; PR, partial response; R, right; (+), positive.

## Data Availability

The data presented in this study are available on request from the corresponding author. The data are not publicly available due to privacy concerns.

## Abbreviations

AEs, adverse events; CR, complete response; F, female; L, left; LNs, lymph nodes; M, male; MM, metastatic melanoma; mm, millimetre; NA, not applicable; NAT, neoadjuvant therapy; OR, operating room; OS, overall survival; pCR, pathological complete response; PR, partial response; R, right; RFS, relapse-free survival; Sx, surgery; TRCs, treatment-related changes; (−), negative; (+), positive.
